# Alterations of Endogenous Hormones, Antioxidant Metabolism, and Aquaporin Gene Expression in Relation to γ-Aminobutyric Acid-Regulated Thermotolerance in White Clover

**DOI:** 10.3390/antiox10071099

**Published:** 2021-07-08

**Authors:** Hongyin Qi, Dingfan Kang, Weihang Zeng, Muhammad Jawad Hassan, Yan Peng, Xinquan Zhang, Yan Zhang, Guangyan Feng, Zhou Li

**Affiliations:** College of Grassland Science and Technology, Sichuan Agricultural University, Chengdu 611130, China; qihongyin@stu.sicau.edu.cn (H.Q.); kangdingfan24@163.com (D.K.); zengwh0123@163.com (W.Z.); jawadhassan3146@gmail.com (M.J.H.); pengyanlee@163.com (Y.P.); zhangxq@sicau.edu.cn (X.Z.); zhangyan1111zy@126.com (Y.Z.); feng0201@sicau.edu.cn (G.F.)

**Keywords:** adaptability, heat stress, water homeostasis, hormone, oxidative damage, stoma, transpiration

## Abstract

Persistent high temperature decreases the yield and quality of crops, including many important herbs. White clover (*Trifolium repens*) is a perennial herb with high feeding and medicinal value, but is sensitive to temperatures above 30 °C. The present study was conducted to elucidate the impact of changes in endogenous γ-aminobutyric acid (GABA) level by exogenous GABA pretreatment on heat tolerance of white clover, associated with alterations in endogenous hormones, antioxidant metabolism, and aquaporin-related gene expression in root and leaf of white clover plants under high-temperature stress. Our results reveal that improvement in endogenous GABA level in leaf and root by GABA pretreatment could significantly alleviate the damage to white clover during high-temperature stress, as demonstrated by enhancements in cell membrane stability, photosynthetic capacity, and osmotic adjustment ability, as well as lower oxidative damage and chlorophyll loss. The GABA significantly enhanced gene expression and enzyme activities involved in antioxidant defense, including superoxide dismutase, catalase, peroxidase, and key enzymes of the ascorbic acid–glutathione cycle, thus reducing the accumulation of reactive oxygen species and the oxidative injury to membrane lipids and proteins. The GABA also increased endogenous indole-3-acetic acid content in roots and leaves and cytokinin content in leaves, associated with growth maintenance and reduced leaf senescence under heat stress. The GABA significantly upregulated the expression of *PIP1-1* and *PIP2-7* in leaves and the *TIP2-1* expression in leaves and roots under high temperature, and also alleviated the heat-induced inhibition of *PIP1-1*, *PIP2-2*, *TIP2-2*, and *NIP1-2* expression in roots, which could help to improve the water transportation and homeostasis from roots to leaves. In addition, the GABA-induced aquaporins expression and decline in endogenous abscisic acid level could improve the heat dissipation capacity through maintaining higher stomatal opening and transpiration in white clovers under high-temperature stress.

## 1. Introduction

Nowadays, the major impact of global warming is already discernible in animal and plant populations [1]. In terms of plants, a global high temperature has resulted in the increase in mortality of forest trees [2], substantial reduction in crop yield [3], and changes in the spatial distribution of herbs [4]. Plant hormones and other plant growth regulators (PGRs) perform pivotal functions in regulating plant adaptability to different abiotic stresses. γ-aminobutyric acid (GABA), a non-protein amino acid comprising four carbons, exists abundantly in living organisms including microbes, plants, and vertebrates. Over the past 50 years, GABA has been regarded as a very important PGR that affects plant growth and the adaptability to high temperature or other environmental stresses [5]. A recent study revealed that the improvement in GABA content or metabolism is an important defense mechanism triggered by the exogenous spermidine (Spd) in white clover (*Trifolium repens*) under heat stress [6]. GABA affected glutathione, carbon, and amino acid metabolism, associated with better heat tolerance in creeping bentgrass (*Agrostis stolonifera*) [7]. Nayyar et al. found that GABA could safeguard rice (*Oryza sativa*) seedlings during high temperature by improving osmotic protection and antioxidant defense [8]. However, study of the regulation mechanisms and physiological function of GABA is still in its infancy as compared to other endogenous hormones (auxin (IAA), gibberellin (GA), cytokinin (CTK), and abscisic acid (ABA)) crucial for plants growth and development [9,10]. As an important signal molecule, ABA participates in the regulation of stomatal movement and multiple gene expression in response to abiotic stress [11]. GA or IAA could effectively alleviate heat-induced growth inhibition and the decline in yield of lemon (*Citrus limon*) trees or rice [12,13]. The CTK-regulated heat tolerance is associated with enhancement of antioxidant defense and delayed leaf senescence in creeping bentgrass [14]. However, it remains unclear so far whether the GABA-regulated heat tolerance is linked with changes in endogenous hormones in plants. 

Oxidative damage is one of the most imperative stress indicators when plants are suffering from heat stress. High temperature leads toward massive production of reactive oxygen species (OH^−^, O_2_^•^^−^, and H_2_O_2_) in plants, resulting in increased oxidative stress to various cell organelles [15]. Plants have evolved an efficient antioxidant defense system (enzymatic and non-enzymatic) to scavenge reactive oxygen species (ROS) under unfavorable environmental conditions. For the enzymatic mechanism, superoxide dismutases (SODs) including Cu/ZnSOD, MnSOD, and FeSOD are the first line of defense against ROS and are involved in the dismutation of superoxide anion to produce hydrogen peroxide and molecular oxygen. The Cu/ZnSOD dominates the main function in higher plants, while the lower plants are dominated by MnSOD and FeSOD [16]. Catalase (CAT), peroxidase (POD), and the ascorbic acid (ASA)–glutathione (GSH) cycle can convert H_2_O_2_ into H_2_O and O_2_. For the ASA–GSH cycle, ascorbate peroxidase (APX), glutathione peroxidase (GPX), monodehydroascorbate reductase (MR), dehydroascorbate reductase (DR), and glutathione reductase (GR) remove H_2_O_2_ through catalyzing the oxidation–reduction of ASA and GSH. In this process, ASA and GSH act as non-enzymatic antioxidants to scavenge ROS [17]. Previous studies showed that the GABA-regulated tolerance under unfavorable conditions was closely related to enhanced antioxidant defense in different plant species. For instance, pepper (*Capsicum annuum*) could resist low-light stress by increasing antioxidant defense after GABA treatment [18], and GABA could slow down the oxidative injury induced by salinity in white clover [19]. However, how GABA interacts with endogenous hormones to affect antioxidant defense systems (enzymatic and non-enzymatic) in leaf and root remains unclear when plants undergo a prolonged period of heat stress. 

Under high-temperature stress, even if water supply is sufficient, plants will suffer from physiological water shortage due to the aggravation of transpiration loss and the decrease in water absorbing capacity in the roots [20]. Water transport in plants is regulated mainly by three ways, including the apoplastic pathway, symplastic pathway, and transcellular pathway. Among them, the transcellular pathway is mainly carried out by aquaporins (AQPs). Four kinds of AQPs have been widely recognized in plants: plasma membrane endogenous proteins (PIPS), vacuolar endogenous proteins (TIPS), nodule protein 26 (NIPS), and small basic endogenous proteins (SIPS) [21]. PIPS are divided into PIP1 and PIP2, that sense the change in water inside and outside of cell membranes, and PIP2 shows a greater water channel efficiency than PIP1. TIPS are located in the vacuolar membrane for sensing the change in water content in the cytoplasm, which performs a vital role in maintaining cell permeability. NIPS is responsible for transporting a wide range of substances, such as water, urea, glycerol, and some metal ions. There are only a few studies on SIPS that are mainly located in endoplasmic reticulum [22,23,24,25]. It was reported that AQPs perform crucial functions in plant water regulation under osmotic or extreme temperature stress. The study of Peng et al. showed that the overexpression of an AQP gene *pgtip1* enhanced the stress tolerance in *Arabidopsis thaliana* under low-temperature, salinity, and water stress [26]. Alexandersson et al. found that *atpip2-2* affected root water transport under drought stress [27]. In soybean (*Glycine max*), *TIP2-6* responds to high-temperature stress through hormone regulation [28]. The expression of *CsT1P1-3*, *CsT1P2-3*, and *CsPIP2-4* was enhanced by high temperature in citrus (*Citrus reticulata Blanco*) [29]. Up to now, it is not well documented whether GABA-regulated thermotolerance is related to AQP expression in plants.

White clover is a perennial herb with high feeding and medicinal value. It is cultivated worldwide as an important forage due to high crude protein content and nutritional value, and extract productions derived from white clover such as flavonoids have multiple effects, such as reducing blood fat, cancer prevention, immunity enhancement, and anti-aging [30,31]. However, white clover is sensitive to high temperature and its growth is inhibited significantly at temperatures above 30 °C [32]. With global warming, the heat-induced damage to white clover is becoming increasingly prominent in summer. The objective of the present study was to determine the impact of GABA on improvement in thermotolerance associated with alterations of endogenous hormones, antioxidant metabolism, water regulation, and AQP-related gene transcript level in leaf and root of white clover. This will help to reveal the potential regulatory mechanism of GABA in herbs under heat stress and improve the cultivation and utilization of white clover or other herbs in more regions.

## 2. Materials and Methods

### 2.1. Plant Materials and Treatments

Seeds of white clover (cultivar “Ladino”) were surface sterilized with 0.1% mercuric chloride (HgCl_2_) solution for 4 min and rinsed thrice with deionized water. Seeds (20 g/m^2^) were placed in a container (24 cm length, 15 cm width, and 8 cm height) comprising quartz sand. The containers were kept in controlled laboratory conditions (23 °C/19 °C day/night, 12 h photoperiod, 700 μmol m^−2^ s^−1^ photosynthetically active radiation, and 75% relative humidity). Seeds were firstly germinated in ddH_2_O for 7 days and then cultured in Hoagland nutrient solution for 24 days [33]. Before the beginning of heat stress, plant materials were grown on Hoagland’s solution with or without 2 mM GABA for 3 days. The half of non-pretreated or GABA-pretreated plants was kept in the controlled growth chamber (conditions as illustrated above) as the control (C) or the control + GABA (C+GABA) for 30 days, and the other half was transferred into a high-temperature chamber (38/33 °C day/night and other conditions the same as the normal growth chamber) as heat stress (H) and heat+GABA (H+GABA) for 30 days. Fresh Hoagland’s solution was applied to the plants every day. The plastic containers were laid out in completely randomized design with four biological replicates. Leaf samples were taken on 0, 5, 10, 15, 20, and 30 d after heat stress, respectively. Root samples were taken at 15 d for gene expression and at 30 d for other parameters.

### 2.2. Determination of Endogenous GABA and Phytohormones Content

The GABA content was estimated by using the Test Kit obtained from Suzhou Comin Biotechnology Co. Ltd, Suzhou, China. following the manufacturer’s guidelines. For the determination of ABA, GA, and IAA, fresh samples (0.4 g) were crushed with 3 mL methanol:isopropanol (1:4, *v*/*v*) and 1% glacial acetic acid. The mixture was centrifuged for 1 h at 4 °C. A total of 2 mL of supernatant was collected, dried, and mixed in CH_3_OH (300 μL). The reaction solution was then filtered by passing through a 0.22 μm PTFE filter [34]. The concentrations of endogenous IAA, GA, and ABA were determined by Waters Acquity UPLCSCIEX Se-lex ION Triple Quad 5500 mass spectrometer (waters, Milford, MA, USA). A 5 μL sample was loaded into the Acquity UPLC beh C18 column (1.7 μm, 50 × 2.1 mm; waters, Waxford, Ireland) at 40 °C. The mobile phase was composed of 40% acetic acid solution and 60% CH_3_OH, and the flow rate was 1 mL min^−1^. The CTK content was estimated by using enzyme-linked immunosorbent assay (ELISA) in accordance with the manufacturer’s guidelines. The Test Kit was obtained from Beijing Fang Cheng Biotechnology Co., Ltd, Beijing, China.

### 2.3. Measurement of Water Status and Photosynthetic Parameters

For leaf relative water content (RWC), the fresh weight (FW) was weighed instantly when leaves were cut off from plants. The leaf samples were drenched in deionized water for 24 h to obtain the saturated weight (SW) and later kept in an oven at 85 °C for 72 h to obtain the dry weight (DW). The RWC was formulated as RWC (%) = [(FW − DW)/(SW − DW)] × 100% [35]. Root viability was estimated by following the procedure of McMichael and Burke [36]. The osmotic potential (OP) was detected by using the protocols of Blum. Fresh leaves or roots were immerged in distilled water for 12 h and quickly frozen in liquid nitrogen for 10 min after the surface moisture being absorbed, and then thawed at 4 °C for 30 min. The cell fluids were squeezed from leaves or roots, and the osmolality of cell sap was determined by using a vapor pressure osmometer (Wescor, Logan, UT, USA), and the OP was converted based on − c × 2.58 × 10^−3^ [37]. For electrolyte leakage (EL), the procedure of Blum and Ebercon was utilized [38]. Chlorophyll (Chl) was measured by Arnon’s method [39]. The photochemical efficiency (Fv/Fm) and performance index absorption basis (PIABS) were measured by using a Chl fluorescence system (Pocket PEA, Hansatech, UK). Prior to analysis, fresh leaf samples were kept in dark conditions for 30 min with attached leaf clips. Net photosynthetic efficiency (Pn), water use efficiency (WUE), stomatal conductance (Gs), intercellular CO_2_ concentration (Ci), and transpiration rate (Tr) were recorded with a movable photosynthetic system (CIRAS-3, PP Systems, Amesbury, MA, USA) that supplied CO_2_ (400 μL^−1^) and red and blue light (800 μmol photon m^−2^), respectively.

### 2.4. Determination of Antioxidant Metabolism

The protein carbonyl content and the total antioxidant capacity (TAC) was measured by using the Test Kit purchased from Suzhou Kangmin Biotechnology Co, Suzhou, China. Superoxide anion (O_2_^•−^) or hydrogen peroxide (H_2_O_2_) were measured following the method of Elstner and Heupel [40] or Velikova et al. [41], respectively. As for malondialdehyde (MDA), SOD, CAT, POD, APX, DR, GR, and MR activities, fresh tissues (0.15 g) were taken and mechanically ground in 1.5 mL of precooled phosphoric acid buffer solution (150 mM and pH 7.0), and then transferred into a centrifugal tube. The homogenate was centrifuged at 15,000× *g* at 4 °C for 20 min to obtain the supernatant. For MDA content, supernatant (0.5 mL) was added in reaction solution (1 mL) having trichloroacetic acid (20%) and thiobarbituric acid (0.5%). The mixture was kept in a boiling water bath (95 °C) for 15 min and later hastily cooled in an ice bath. The solution was centrifuged at 8000× *g* for 10 min at 4 °C. The absorbance value was noticed spectrometrically at 532 and 600 nm [42]. The activities of SOD, CAT, POD, or APX were estimated by noting absorbance values at 560, 240, 470, or 290 mm, respectively [43,44,45]. The GR, MR, or DR activity was detected by using the protocols of Cakmak et al. [46]. Bradford’s method was used to detect soluble protein content [47]. ASA and GSH content were measured using the method of Gossett et al. [48].

### 2.5. Total RNA Extraction and qRT-PCR Analysis

Real time quantitative polymerase chain reaction (qRT-PCR) was used to determine the gene expression. The Rneasy Mini Kit (Qiagen, Duesseldorf, Germany) was used for extracting total RNA in fresh leaves or roots. The RNA samples were treated with DNAsa to remove possible DNA and then reverse-transcribed to cDNA using a revert Aid First Stand cDNA Synthesis Kit (Fermentas, Lithuania). Primers of antioxidant enzyme genes (*Cu/ZnSOD*, *MnSOD*, *FeSOD*, *CAT*, *POD*, *APX*, *DR*, *GR*, *MR*) and aquaporin genes (*PIP1-1*, *PIP2-2*, *PIP2-7*, *SIP1-1*, *TIP1-1*, *TIP2-1*, *TIP2-2*, *NIP1-2*, *NIP2-1*) are recorded in Table S1 (*β*-actin as an internal control). For all genes, PCR conditions (iCycler iQ qRT-PCR detection system with SYBR Green Supermix, Bio-Rad, Hercules, CA, USA) were as follows: 5 min at 94 °C and 30 s at 95 °C (45 repeats of denaturation), annealing and extending for 45 s at 58 to 66 °C (Table S1), and amplicon from 60 to 95 °C to obtain the melting curve.

### 2.6. Statistical Analysis 

Statistical analysis of all the data was conducted using SPSS 23 (IBM, Armonk, NY, USA). Significant differences among various treatments were estimated by one-way ANOVA together with LSD test at 5% probability level (*p* ≤ 0.05).

## 3. Results

### 3.1. Changes in Endogenous GABA and Hormones Content

**Figure 1:**
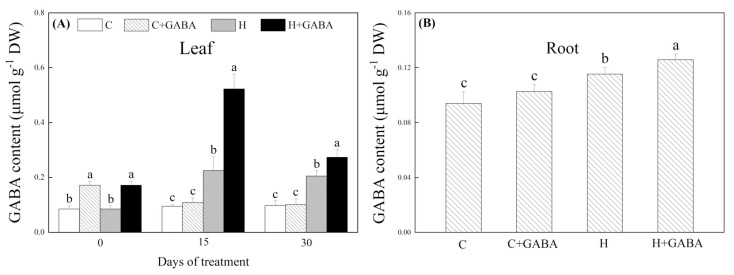
Effects of GABA application on the content of GABA in plant leaves (**A**) and roots (30th day) (**B**) under normal-temperature and high-temperature stress. The vertical bar above the column in (**A**) or (**B**) represents + SE of the mean (*n* = 4) and the different letters in the columns indicate significant differences at a given day of treatment based on LSD (*p* ≤ 0.05). C, control (normal condition); C + GABA, control plants pretreated with GABA (normal condition); H, heat stress; H + GABA, heat-stressed plants pretreated with GABA.

**Figure 2:**
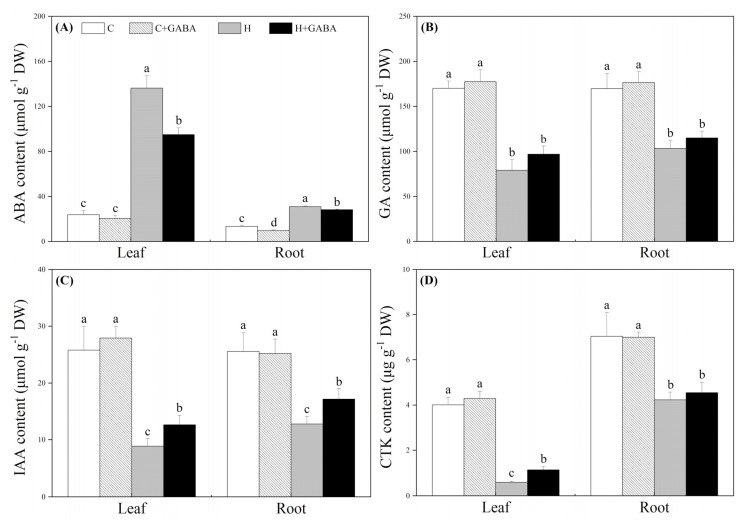
Effects of GABA application on ABA content (**A**), GA content (**B**), IAA content (**C**), and CTK content (**D**) of leaves (30th day) and roots (30th day) under normal-temperature and high-temperature stress. The vertical bar above the column in (**A**), (**B**), (**C**), or (**D**) represents + SE of the mean (*n* = 4) and the different letters in the column indicate significant differences in leaf or root based on LSD (*p* ≤ 0.05). C, control (normal condition); C + GABA, control plants pretreated with GABA (normal condition); H, heat stress; H + GABA, heat-stressed plants pretreated with GABA.

The endogenous GABA content significantly increased after exogenous GABA pretreatment (Figure 1A). On the 15th day, the GABA content increased significantly in the GABA-pretreated and non-pretreated plants under the high-temperature condition, and the increase was more significant in the GABA-pretreated plants (Figure 1A). On the 30th day, the GABA content in the roots and leaves of two heat-stressed treatments (“H” and “H + GABA”) was still significantly higher in contrast to normal treatments (“C” and “C + GABA”), and GABA pretreatment could further increase heat-induced GABA content in roots and leaves (Figure 1A,B). The GABA pretreatment had no significant effect on ABA, GA, IAA, and CTK contents in leaves and roots under normal conditions (Figure 2A–D). The ABA content increased significantly, but GA, IAA, and CTK contents decreased significantly in leaves and roots after 30 d of heat stress (Figure 2A–D). Under the high-temperature condition, the GABA application significantly inhibited heat-induced increases in ABA content in roots and leaves (Figure 2A), but did not affect the GA content (Figure 2B). The IAA content in roots and leaves as well as CTK content in leaves were increased significantly by the GABA pretreatment (Figure 2C,D).

### 3.2. Effects of GABA on Cell Membrane Stability and Oxidative Damage

**Figure 3:**
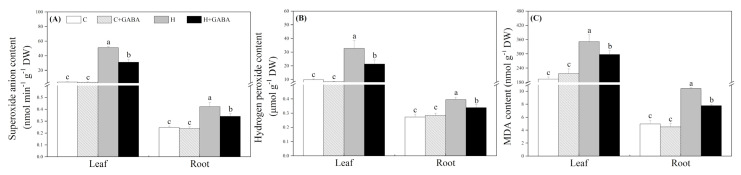
Effects of GABA application on superoxide anion content (**A**), hydrogen peroxide content (**B**), and malondialdehyde (MDA) content (**C**) of plants leaves (30th day) and roots (30th day) under normal-temperature and high-temperature stress. The vertical bar above the column in (**A**), (**B**), or (**C**) represents + SE of the mean (*n* = 4) and the different letters in the column indicate significant differences in leaf or root based on LSD (*p* ≤ 0.05). C, control (normal condition); C + GABA, control plants pretreated with GABA (normal condition); H, heat stress; H + GABA, heat-stressed plants pretreated with GABA.

**Figure 4:**
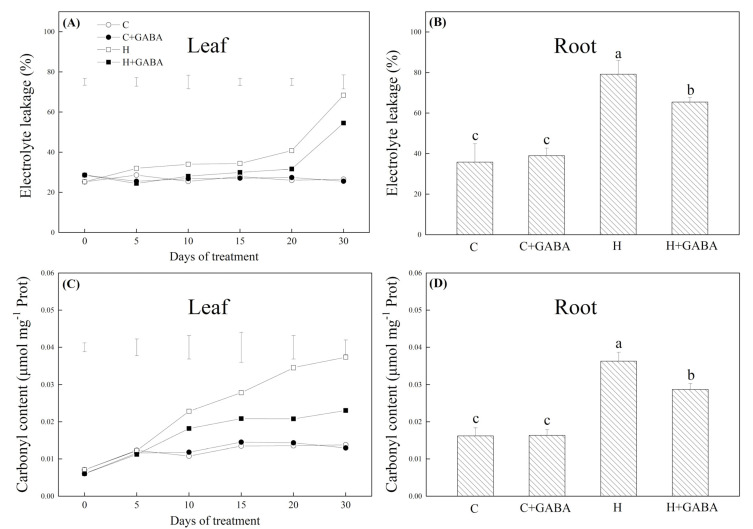
Effects of GABA application on electrolyte leakage of leaves (**A**) or roots (30th day) (**B**), carbonyl content of leaves (**C**) or roots (30th day) (**D**) of plants under normal-temperature and high-temperature stress. Vertical bars above curves in (**A**) or (**C**) represent the least significant difference (LSD) values at a particular day (*n* = 4; *p* ≤ 0.05). The vertical bar above the column in (**B**) or (**D**) represents + SE of the mean (*n* = 4) and the different letters in the columns indicate significant differences based on LSD (*p* ≤ 0.05). C, control (normal); C + GABA, control plants pretreated with GABA; H, heat stress; H + GABA, heat-stressed plants pretreated with GABA.

The MDA, H_2_O_2_, and O_2_^•−^ content significantly increased in leaves and roots of the GABA-treated and untreated plants due to high-temperature stress and the GABA application could significantly alleviate these effects (Figure 3A–C). However, no significant differences in the accumulation of O_2_^•−^, H_2_O_2_, and MDA content between GABA- treated and untreated plants were observed under normal conditions (Figure 3A–C). EL in leaves gradually increased with the extension of heat stress time. The GABA-pretreated plants maintained a 12.86, 22.48, or 20.27% decrease in EL in leaves as compared to the non-pretreated plants on 15, 20, or 30 d of high-temperature stress, respectively (Figure 4A). Similarly, exogenous GABA application also effectively alleviated the heat-induced increase in EL in roots after 30 d (Figure 4B). With the passage of stress time, the carbonyl content increased significantly under high temperature, but the application of GABA showed a significant inhibitory effect on the increase in carbonyl content in leaves and roots in response to heat stress (Figure 4C,D). The GABA-treated white clover plants showed a significant reduction in carbonyl content when compared to untreated plants on the 15th, 20th, and 30th days of heat stress (Figure 4C). Under heat stress, the GABA-pretreated plants maintained a 20.86% greater decrease in the carbonyl content than non-pretreated plants in roots (Figure 4D).

### 3.3. Effect of GABA on Antioxidant Metabolism

**Figure 5:**
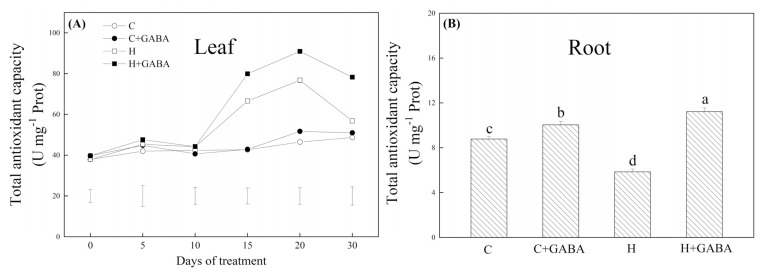
Effects of GABA application on total antioxidant capacity of leaves (**A**) or roots (30th day) (**B**). Vertical bars above curves in (**A**) represent the least significant difference (LSD) values at a particular day (*n* = 4; *p* ≤ 0.05). The vertical bar above the column in (**B**) represents + SE of the mean (*n* = 4) and the different letters in the columns indicate significant differences based on LSD (*p* ≤ 0.05). C, control (normal condition); C + GABA, control plants pretreated with GABA (normal condition); H, heat stress; H + GABA, heat-stressed plants pretreated with GABA.

**Figure 6:**
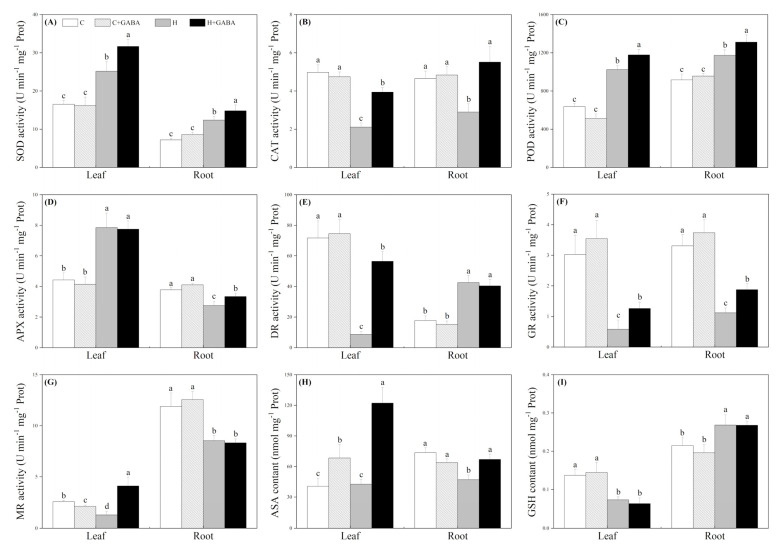
Effects of GABA application on SOD content (**A**), CAT content (**B**), POD content (**C**), APX content (**D**), DR content (**E**), GR content (**F**), MR content (**G**), ASA content (**H**), and GSH content (**I**) of plant leaves (30th day) and roots (30th day) under normal-temperature and high-temperature stress. The vertical bar above the column in (**A**–**I**) represents + SE of the mean (*n* = 4) and the different letters in the column indicate significant differences in leaf or root based on LSD (*p* ≤ 0.05). C, control (normal condition); C + GABA, control plants pretreated with GABA (normal condition); H, heat stress; H + GABA, heat-stressed plants pretreated with GABA.

The TAC in leaves of the GABA-pretreated and non-pretreated plants gradually increased from 10 to 20 d of heat stress and then declined on the 30th day of heat stress (Figure 5A). The GABA pretreatment did not greatly influence the TAC in leaves under normal condition, but the GABA-pretreated plants exhibited 20.03, 18.39, or 37.75% higher TAC compared to the non-pretreated plants on the 15th, 20th, or 30th day of heat stress, respectively (Figure 5A). In the root system, the TAC in the heat-treated plants was significantly lower in contrast to the control group “C”, but with the application of GABA, the TAC in roots increased significantly under heat stress (Figure 5B). Under normal conditions, the GABA pretreatment also significantly improved the TAC in roots (Figure 5B). For changes in antioxidant enzyme activities, heat stress significantly improved SOD and POD activities in leaves and roots, and the GABA-pretreated plants showed significantly higher SOD and POD activities in leaves and roots compared with the non-pretreated plants under high temperature (Figure 6A,C). Under heat stress, the GABA-pretreated plants maintained an 86.81 or 90.14% increase in CAT activity in leaves or roots than non-pretreated plants, respectively (Figure 6B). Heat stress significantly improved APX activity in leaves and DR activity in roots, but the GABA pretreatment did not significantly affect APX activity in leaves and DR activity in roots under normal as well as stressed conditions (Figure 6D,E). However, the GABA pretreatment effectively alleviated heat-induced declines in AXP activity in roots, DR activity in leaves, and GR activities in leaves and roots (Figure 6D–F). The GABA-pretreated plants showed a 217.05% increase in MR activity in leaves compared to non-pretreated white clover plants subjected to heat stress (Figure 6G). In leaves, ASA content was substantially ameliorated by the exogenous application of GABA under normal as well as stressed conditions. In roots, the GABA application could also stop the heat-caused decline in ASA content (Figure 6H). GSH content significantly declined in leaves and was enhanced in roots under heat stress; however, the GABA application did not exert its significant effects on GSH content under any of the above-mentioned treatments (Figure 6I). 

**Figure 7:**
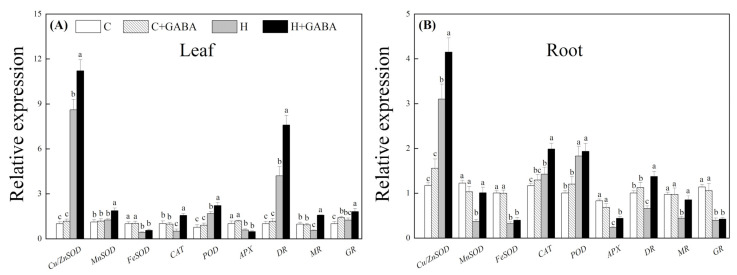
Effect of GABA on relative expression of *Cu/ZnSOD, MnSOD, FeSOD, CAT, POD, APX, DR, MR*, and *GR* genes in leaves (15th day) (**A**) and roots (15th day) (**B**). The vertical bar above the column in (**A**) and (**B**) represents + SE of the mean (*n* = 4) and the different letters in the columns indicate significant differences based on LSD (*p* ≤ 0.05). C, control (normal condition); C + GABA, control plants pretreated with GABA (normal condition); H, heat stress; H + GABA, heat-stressed plants pretreated with GABA.

Heat stress significantly upregulated the expression level of *Cu/ZnSOD*, *POD*, and *DR* in leaves treated with or without GABA (Figure 7A). A 30.34, 32.50, or 81.14% increase in *Cu/ZnSOD*, *POD*, or *DR* expression was observed in leaves of the “H + GABA” as compared to that of the “H”. The *MnSOD* expression in leaves was only significantly improved in the GABA-pretreated plants by heat stress. The *FeSOD* and *APX* expression significantly decreased in leaves under heat stress, and there were no significant differences in *FeSOD* expression between the GABA-treated and untreated plants under control as well as stressed conditions (Figure 7A). *CAT* and *MR* expression in leaves showed similar results in response to the GABA pretreatment and heat stress, as reflected by a significant decline in the non-pretreated plants and a significant increase in the GABA-pretreated plants under heat stress. The GABA pretreatment significantly enhanced *GR* expression in leaves under control and stressed conditions (Figure 7A). For gene expression in roots, the GABA-treated plants exhibited significantly higher *Cu/ZnSOD*, *MnSOD*, *CAT*, *APX*, *DR*, and *MR* than the non-pretreated plants under heat stress (Figure 7B). In addition, heat stress significantly inhibited the *FeSOD* and *GR* expression and improved *POD* expression in roots of the GABA-pretreated and non-pretreated plants, but the GABA pretreatment did not show any significant impact on the expression of these genes under control as well as heat-stress conditions (Figure 7B). 

### 3.4. Effects of GABA on Photosynthesis and Water Status

**Figure 8:**
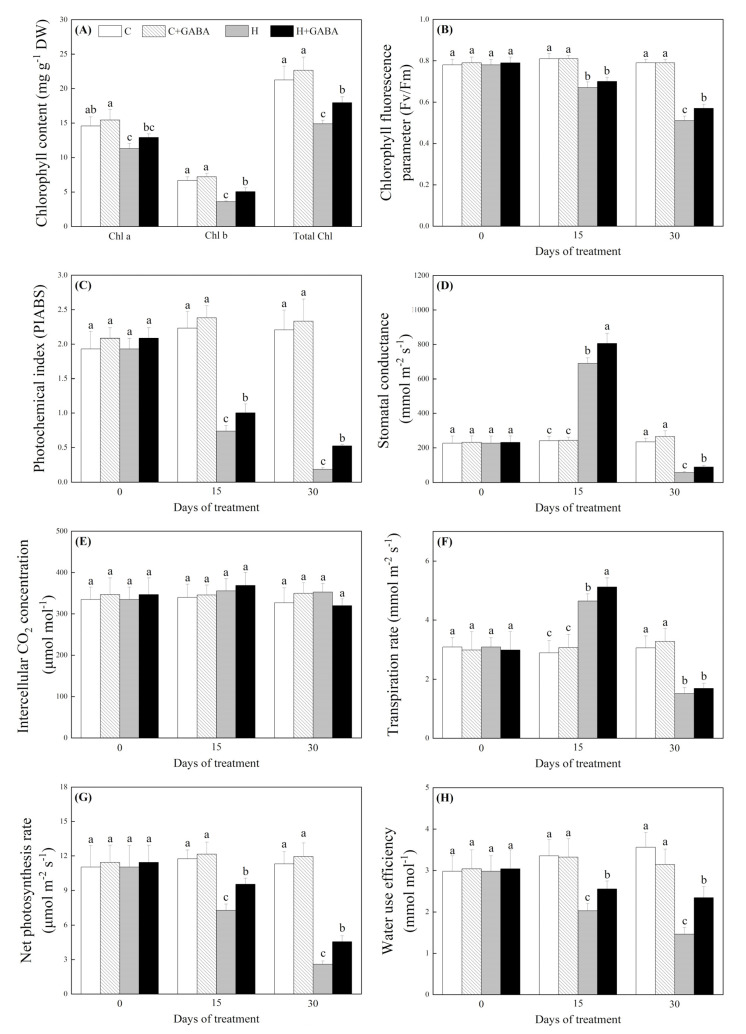
Effects of GABA application on chlorophyll a, b, or total content (**A**), chorophyll fluorescence parameter (**B**), photochemical index (**C**), stomatal conductance (**D**), intercellular CO_2_ concentration (**E**), transpiration rate (**F**), net photosynthesis rate (**G**), or water use efficiency (**H**) of plants under normal-temperature and high-temperature stress. The vertical bar above the column in (**A**) to (**H**) represents + SE of the mean (*n* = 4) and the different letters in the columns indicate significant differences at a given day of treatment based on LSD (*p* ≤ 0.05). C, control (normal condition); C + GABA, control plants pretreated with GABA (normal condition); H, heat stress; H + GABA, heat-stressed plants pretreated with GABA.

**Figure 9:**
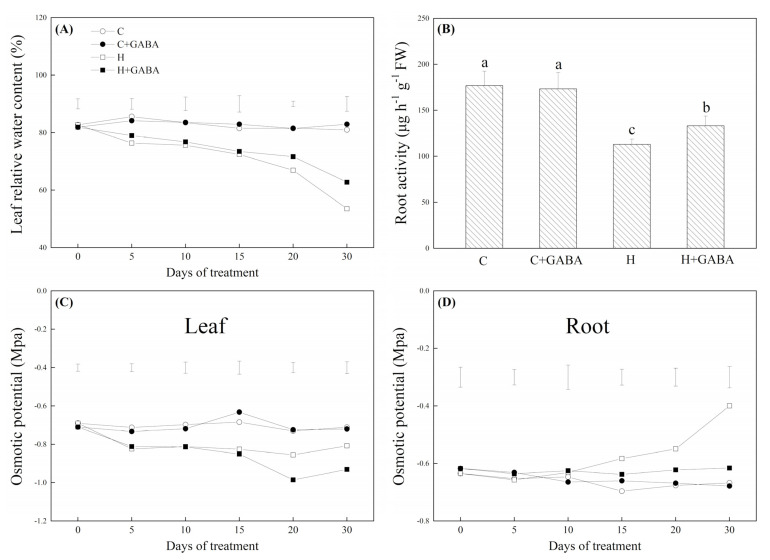
Effects of GABA application on leaf relative water content (**A**), root activity (30th day) (**B**), and osmotic potential of leaves (**C**) or of roots (**D**) of plants under normal-temperature and high-temperature stress. Vertical bars above curves in (**A**), (**C**), or (**D**) represent the least significant difference (LSD) values at a particular day (*n* = 4; *p* ≤ 0.05). The vertical bar above the column in (**B**) represents + SE of the mean (*n* = 4) and the different letters in the columns indicate significant differences based on LSD (*p* ≤ 0.05). C, control (normal condition); C + GABA, control plants pretreated with GABA; H, heat stress (normal condition); H + GABA, heat-stressed plants pretreated with GABA.

Chl a, Chl b, total Chl content, Fv/Fm, PIABS, Pn, and WUE significantly decreased under heat stress, but the application of GABA significantly alleviated their declines under heat stress (Figure 8A–C,G,H). Upon exposure to high temperature, Gs and Tr increased significantly on the 15th day, but decreased on the 30th day. GABA induced significant increases in Gs on the 15th and 30th days of heat stress and Tr on the 15th day of heat stress (Figure 8D,F). The Ci was not changed significantly by heat stress and the GABA application (Figure 8E). WUE and Tr showed a significant downward trend during heat stress, but the downward trend was significantly alleviated by GABA application (Figure 8G,H). Leaf RWC, root activity, and OP in leaf and root did not show any significant difference by foliar application of GABA under controlled conditions (Figure 9A–D). Under heat stress, the leaf RWC and root activity declined markedly in GABA treated and untreated white clover plants, but the GABA-pretreated plants exhibited a 17.23% or 22.73% greater increase in leaf RWC or root activity than non-pretreated plants on the 30th day of heat stress, respectively (Figure 9A,B). High-temperature stress significantly reduced OP in leaves, while it increased OP in roots (Figure 9C,D). The GABA pretreatment further decreased heat-induced decline in OP in leaves on the 20th and 30th days of heat stress (Figure 9C). In addition, the GABA-pretreated plants also demonstrated a 15.32% or 15.22% greater decline in OP in roots than the non-pretreated plants on the 20th and 30th days of heat stress, respectively (Figure 9D). 

### 3.5. Effect of GABA on AQP Gene Expression

**Figure 10:**
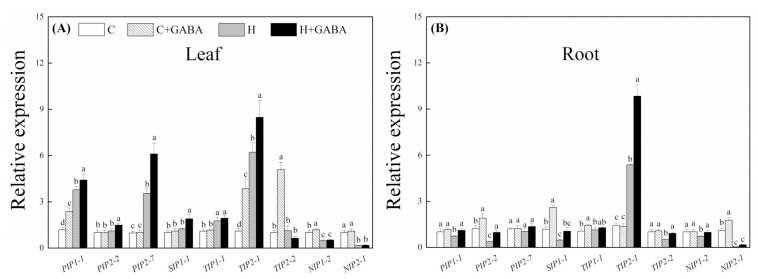
Effect of GABA on relative expression of *PIP1-1, PIP2-2, PIP2-7, SIP1-1, TIP1-1, TIP2-1, TIP2-2, NIP1-2*, and *NIP2-1* genes in leaves (15th day) (**A**) and roots (15th day) (**B**). The vertical bar above the column in (**A**,**B**) represents + SE of the mean (*n* = 4) and the different letters in the column indicate significant differences based on LSD (*p* ≤ 0.05). C, control (normal condition); C + GABA, control plants pretreated with GABA (normal condition); H, heat stress; H + GABA, heat-stressed plants pretreated with GABA.

For the transcription level of genes encoding AQPs, heat stress significantly upregulated the transcript level of *PIP1-1*, *PIP2-7*, *TIP1-1*, and *TIP2-1* in leaves of the GABA-pretreated and untreated plants (Figure 10A). As compared with the “H” treatment, the transcript level of *PIP1-1*, *PIP2-7*, or *TIP2-1* in the “H + GABA” treatment increased by 10.81, 74.28, or 35.48%, respectively. Under heat stress, the expression of *NIP1-2* and *NIP2-1* in leaves decreased significantly, and no significant difference between the GABA-treated and untreated plants was observed. As compared to the “C” treatment, the expression of *SIP1-1* did not change significantly in the “H” treatment under heat stress, but increased by 52.41% in the “H + GABA” treatment (Figure 10A). For AQP gene expression in roots, heat stress significantly inhibited the expression of *PIP1-1*, *PIP2-2*, *SIP1-1*, *TIP2-2*, *NIP1-2*, and *NIP2-1* in plants without the GABA pretreatment. However, the GABA-pretreated plants maintained a significantly higher expression of *PIP1-1*, *PIP1-2*, *PIP2-1*, *SIP1-1*, *TIP2-1*, *TIP2-2*, and *NIP2-1* when compared with non-pretreated plants under high-temperature stress (Figure 10B). The GABA application further upregulated the heat-induced increase in *TIP2-1* expression in roots (Figure 10B).

## 4. Discussion

When plants are exposed to high temperatures, GABA as a metabolite or signal molecule regulates intracellular pH environment, carbon (C)–nitrogen (N) nutrient metabolism, oxidative and osmotic balance, and signal transmission [49]. In this study, the GABA pretreatment not only further enhanced the heat-induced GABA content in roots and leaves of white clover, but also significantly alleviated Chl loss and declines in Pn and Fv/Fm under high temperature. A previous study proved that the increase in endogenous GABA could improve the heat resistance of plants such as creeping bentgrass, perennial ryegrass (*Lolium perenne*), and vegetable soybean (*Glycine max*) [50,51,52]. Under unfavorable environmental conditions, changes in endogenous hormones are critical modulatory factors of stress tolerance in plants [53]. The study of Lancien and Roberts indicated that potential interactions between GABA, ABA, and ethylene modulated key genes involved in C–N metabolism in *Arabidopsis thaliana* under normal conditions [54]. Enhanced GABA accumulation induced by exogenous GABA application in roots of maize (*Zea mays*) were accompanied by a substantial reduction in ABA content and increment in GA, IAA, and CTK content during saline conditions [55]. Our present findings demonstrate that exogenous GABA significantly inhibited ABA accumulation in leaves and roots, and improved IAA content in leaves and roots and CTK content in leaves of white clover, which implied that the GABA-mediated tolerance under extreme temperature was linked with changes in ABA, GA, and IAA. Stress-induced increase in ABA content activates defensive responses, including stomatal closure, defensive gene expression, and protein accumulation [56,57]. However, stomatal closure is unfavorable for transpiration and thermolysis. The IAA accumulation in roots promotes the development of lateral roots, thus enhancing the water absorption performance of *Arabidopsis thaliana* [58], and can also improve the heat tolerance of rice [12]. Among the most pivotal roles of CTK in leaves is to delay leaf senescence by inhibiting Chl loss, membrane deterioration, and protein degradation [59]. Maintenance of higher CTK levels was proved to be of importance for heat tolerance [60].

High temperature caused a large amount of ROS accumulation which caused damage to proteins and membrane lipids [61]. Beneficial effects of GABA in antioxidant enzyme activities and ROS scavenging have been exhibited in leaves of different plant species under water-deficient conditions, salt, and high-temperature stress [62,63,64], but relatively little research has been conducted to discuss the effects of GABA in antioxidant metabolism in roots under abiotic stress. The current findings show that high-temperature stress results in ROS (O_2_^•−^ and H_2_O_2_) accumulation and oxidative damage to membrane lipids or proteins, as shown by the considerable upsurge in MDA content or protein carbonyl content in roots and leaves of white clover. For comparing the difference in oxidative damage between leaves and roots, it can be seen that the leaves suffered more severe oxidative damage than roots after the same duration of heat stress, which is consistent with the earlier research on bermudagrass (*Cynodon dactylon*) [65]. In order to minimize ROS damage, the antioxidant defense was comprehensively elevated during heat stress [66]. After 30 d of heat stress, white clover plants pretreated with GABA exhibited significantly higher TAC, SOD, CAT, and POD activities in roots and leaves than untreated plants. For the ASA–GSH cycle, exogenous GABA promoted APX and GR activities as well as ASA content in roots and leaves during high-temperature stress. The ASA is one of the most crucial non-enzymatic antioxidants for ROS scavenging and the maintenance of its content also plays a key role in the ASA–GSH cycle [67,68]. The study of Li et al. reported that GABA ameliorated the ASA–GSH cycle in favor of thermotolerance in creeping bentgrass, which is a heat-sensitive perennial grass [62]. Interestingly, DR and MR activities were only significantly improved by the GABA in leaves, but not in roots of white clover under heat stress. This could indicate that the GABA-regulated differential antioxidant enzymes might be dependent on the severity of stress, since the leaves suffered from more severe oxidative damage. In terms of genes involved in antioxidant metabolism, the *Cu/ZnSOD* expression in leaves and roots was extremely enhanced by heat stress and the GABA application, reflecting its more imperative function in white clover under heat stress as compared to *MnSOD* and *FeSOD*. It was found that *Cu/ZnSOD* dominates the main function in higher plants [69]. The GABA-regulated genes encoding antioxidant enzymes were consistent with the enzyme activities under heat stress. These findings indicate that GABA helped to maintain antioxidant metabolism in leaves and roots of white clover through regulating enzyme activities and gene expression under heat stress.

In addition to oxidative damage and photosynthetic damage, plants also suffer physiological drought, which often appears in the later period of heat stress due to increased transpiration and root death, leading to a decline in water uptake, even if the water supply is adequate in soil [20]. This could explain why the leaves RWC in white clover slightly decreased with the development of heat stress and then declined sharply after 20 d of heat stress. Different from drought stress, ABA was not significantly induced in the early stage of heat stress [70], which means that stomata will remain open to increase heat dissipation in creeping bentgrass. At the later stage of heat stress, the plants synthesized ABA to regulate stomatal closure in order to decrease the transpiration rate [71]. Our results demonstrate that white clover plants pretreated with GABA exhibited significantly higher stomatal conductance and transpiration rate, which could be contributed to better thermolysis of white clover under heat stress. This might be linked with inhibition of ABA accumulation induced by the GABA in white clover. In addition, the maintenance of higher root activity, Pn, and leaf WUE was observed in the GABA-pretreated white clover. The WUE reflects the homeostasis between transpiration and Pn, which is good for adaptation under water-deficient conditions. Kumar et al. reported that the GABA-promoted WUE helped to keep water homeostasis in plants under abiotic stress [72]. Higher root activity is propitious to water and nutrient transport in plants upon exposure to harsh environments [73]. Heat stress also induced continual decline in OP in leaves, but an increase in OP in roots in both GABA-pretreated and non-pretreated white clover, demonstrating different responses to heat stress between leaves and roots. However, the GABA-pretreated white clover exhibited significantly lower OP in leaves and roots than untreated plants during the late stage of heat stress (20 and 30 d) associated with higher RWC in leaves of the GABA-pretreated white clover. Our earlier findings in creeping bentgrass demonstrate that GABA enhanced organic metabolites accumulation (amino acids and sugars) leading to the decline in OP, thereby improving water balance in leaves under elevated temperatures, salinity, and water-deficient conditions [74]. 

The GABA application further upregulated heat-induced increases in *PIP1-1* and *PIP2-7* expression in leaves and *TIP2-1* expression in leaves and roots of white clover under high-temperature stress. According to previous studies, the main function of PIP and TIP was to transport water molecules through cell or vacuole membranes, which is very important to maintain water uptake and homeostasis of cells [75,76,77]. Forrest and Bhave also proved that PIP and TIP as key AQPs had a strong function of water regulation against osmotic stress in wheat [76]. The spermine increased the *PIP2-7* expression and PIP2-7 accumulation was associated with better water transportation and balance in white clover and *Arabidopsis* during water shortage [77]. *TIP2-1* is considered to be an important pressure-gated water channel in grapevine (*Vitis vinifera*) subjected to various abiotic stresses [78]. Moreover, heat stress did not significantly affect *PIP2-2* and *SIP1-1* expression in leaves of white clover, but the GABA transcripts for these two genes under heat stress. Interestingly, the GABA effectively alleviated heat-induced inhibition of *PIP1-1*, *PIP2-2*, *TIP2-2*, and *NIP1-2* expression in roots of white clover. In the previous study of Grondin et al., the expression of *PIP1-1* and *PIP2-2* was correlated with water flux in roots [79]. The study of Liu et al. also proved that the overexpression of *PIP2-2* in roots could increase the osmotic adjustment ability of plants under salt stress [80]. Na^+^ induced PIP1 and TIP1 accumulation and gene expression in roots and leaves of white clover, which could enhance water transport under drought stress [81]. These findings indicated that the GABA-regulated tolerance in white clover might be related with increases in water transport and homeostasis through activating AQPs expression in leaves and roots under heat stress.

Apart from the main function of AQPs for transporting H_2_O or various molecules, including CO_2_, H_2_O_2_, and (NH_2_)_2_CO, across cells, AQPs also play a vital role in the regulation of stomatal conductance and transpiration in plants [82,83,84]. The study of Lin et al. found that *Arabidopsis* plants overexpressing *PgTIP1* showed significantly higher stomatal conductance and transpiration rate in contrast to the wild type [85]. Plant vigor and transpiration rate were also significantly improved due to the overexpression of a *PIP1b* in transgenic tobacco (*Nicotiana tabacum*) under normal conditions [86]. In addition, constitutive expression of a *TIP2-2* in transgenic tomato (*Lycopersicon esculentum*) plants increased water mobility and transpiration under well-watered as well as drastic environmental conditions [87]. On the contrary, the stomatal conductance and transpiration could be inhibited significantly due to the downregulation of AQP expression in various plant species [88,89,90]. Our current findings show that white clover increased transpiration through increasing stomatal conductance in favor of the improvement in heat dissipation on the 15th day of high-temperature stress. Interestingly, stomatal conductance and transpiration were further increased in leaves of white clover because of the further increase in endogenous GABA level by the exogenous GABA application. The variation tendency of stomatal conductance and transpiration was consistent with the AQP gene expression regulated by GABA in white clover under heat stress. Our findings could indicate that the GABA-upregulated AQP gene expression could contribute to better heat dissipation performance related to stomatal opening and transpiration under high-temperature stress. 

## 5. Conclusions

The improvement in endogenous GABA in leaf and root by GABA pretreatment could significantly alleviate the damage to white clover during high-temperature stress, as demonstrated by enhancements in cell membrane stability, photosynthetic capacity, and osmotic adjustment ability, as well as lower oxidative damage and Chl loss. The GABA significantly upregulated the transcript levels of antioxidant enzyme genes, and improved antioxidant enzyme activity (SOD, CAT, and POD) and key enzymes involved in the ASA–GSH cycle, thus reducing the oxidative damage to membrane lipids and proteins. In terms of plant hormones, GABA increased IAA content in roots and leaves and CTK content in leaves, associated with growth maintenance and reduced leaf senescence under heat stress. GABA upregulated the expression of *PIP1-1* and *PIP2-7* in leaves and *TIP2-1* expression in leaves and roots under high temperatures, and also alleviated the heat-induced inhibition of the expression of *PIP1-1*, *PIP2-2*, *TIP2-2*, and *NIP1-2* in roots. This could help to improve the water transportation and homeostasis under heat stress. In addition, GABA induced AQP expression and a decline in endogenous ABA levels, which could enhance the heat dissipation capacity through maintaining higher stomatal opening and transpiration in white clovers under high-temperature stress.

## Data Availability

Data are contained within the article and Supplementary Materials.

## Supplementary Materials

The following are available online at https://www.mdpi.com/article/10.3390/antiox10071099/s1, Table S1: Primer sequences used in qRT-PCR.
