# Supplementary material for: Alterations of Endogenous Hormones, Antioxidant Metabolism, and Aquaporin Gene Expression in Relation to γ-Aminobutyric Acid-Regulated Thermotolerance in White Clover
Source: Antioxidants (Basel). 2021 Jul 8;10(7):1099. doi: 10.3390/antiox10071099 (PMC8301151; doi:10.3390/antiox10071099)
Supplement: Supplementary file 1 [file antioxidants-10-01099-s001.zip › antioxidants-1277846-supplementary.pdf]

Table S1. Primer sequences used in qRT-PCR.

Target gene	Accession No.	Forward primer (5'-3')	Reverse primer (5'-3')	Tm/℃
Cu/ZnSOD	JQ321597.1	AACTGTGTACCACGAGGACTTC	AGACTAACAGGTGCTAACAACG	58
FeSOD	KP202173	ACACGATTTCTCAGGGTTACGAC	GCGGCCAAGACTATCAGTTCCAT	58
MnSOD	JQ321598.1	TAAGGGAACCTACCCGATAACT	CCAGGACCAAACGTCACCAAAG	66
CAT	JQ321596.1	AACAGGACGGGAATAGCACG	ACCAGGTTCAGACACGGAGACA	58
POD	JQ321606.1	CACTTGGTTTAGTTTTGTCGCC	AACACGGTCTTGTCTGCTACG	64
APX	JQ321599.1	TAAAGATAGTCAACCCACCTCAACA	ACCAGTCTTGGGAAACAACGTA	58
MR	KP202172	CCAACTGCCTAAAGCCACATCT	GAAGAAAGGAAACTAACGGAGCAT	64
DR	KP202171	TGGTTACCTCCCGACCCTAT	TCTTACCAAGGAACTTTAGTCAGG	58
GR	JQ321602.1	TAAACTTCCACTCCCTTTCTATCG	CTACAATATGGGTTGAGGACAGGT	58
PIP1-1	MF405356	GTTCTTGTCTACACCGTATTCTCCG	CCAGTTCCAGTGATTGGGATAGTG	57
PIP2-2	MF405358	TGAAACACCCTTACAACCACCTC	TGCGCTTCTCTTTGGATCAGTAG	59
PIP2-7	MF405359	TGATGGTTACAACAAAGGTACAGCT	AATCGGGATAGTAGCCAAGTGAA	57
SIP1-1	MF405360	CTACCTGGCTTATTCATCACAACAA	CGCCTATTGCTTGAGCTGGA	57
TIP1-1	AJ243309.1	AGTGGGTCCCGCTTTGGTGT	CATGGATGCTCCGGTGAAGG	57
TIP2-1	MF405362	AGATGCAGCACTTGATCCAGC	CCAAAGGTGACAGCAGGGTTA	57
TIP2-2	MF405363	GGAGTGGCTGCTGGATTGAA	ATGGACCGGCTGCTAAGATG	57
NIP1-2	KU598952	TGAACCCAGTTAGAAGCCTAGGAC	TCACGAACGGACTTGTTAGTGTATC	61
NIP2-1	MF405354	GTGCACACATGAATCCGGCT	CTTTGATGGTTCTAACAACGCTCTA	57
β-Actin	JF968419	TTACAATGAATTGCGTGTTG	AGAGGACAGCCTGAATGG	58
